# Comparative genomics of the classical *Bordetella* subspecies: the evolution and exchange of virulence-associated diversity amongst closely related pathogens

**DOI:** 10.1186/1471-2164-13-545

**Published:** 2012-10-10

**Authors:** Jihye Park, Ying Zhang, Anne M Buboltz, Xuqing Zhang, Stephan C Schuster, Umesh Ahuja, Minghsun Liu, Jeff F Miller, Mohammed Sebaihia, Stephen D Bentley, Julian Parkhill, Eric T Harvill

**Affiliations:** 1 Department of Veterinary and Biomedical Sciences, The Pennsylvania State University, University Park, PA, USA; 2 Graduate Program in Bioinformatics and Genomics, The Pennsylvania State University, University Park, PA, USA; 3 Department of Biochemistry and Molecular Biology, The Pennsylvania State University, 310 Wartik Laboratory, University Park, PA, USA; 4 Graduate Program in Genetics, The Pennsylvania State University, University Park, PA, USA; 5 Department of Microbiology, Immunology and Molecular Genetics, University of California, Los Angeles, CA, USA; 6 Wellcome Trust Sanger Institute Hinxton, Cambridge, UK; 7 Singapore Centre on Environmental Life Sciences Engineering, Nanyang Technical University, Nanyang, Singapore; 8 Department of Biology, University Hassiba Ben-Bouali de Chlef, Chlef, Algeria

**Keywords:** Genome, Bordetella, SNP, Pan-genome, Virulence, Evolution, Horizontal gene transfer, Host adaptation, Pertussis toxin

## Abstract

**Background:**

The classical *Bordetella* subspecies are phylogenetically closely related, yet differ in some of the most interesting and important characteristics of pathogens, such as host range, virulence and persistence. The compelling picture from previous comparisons of the three sequenced genomes was of genome degradation, with substantial loss of genome content (up to 24%) associated with adaptation to humans.

**Results:**

For a more comprehensive picture of lineage evolution, we employed comparative genomic and phylogenomic analyses using seven additional diverse, newly sequenced *Bordetella* isolates. Genome-wide single nucleotide polymorphism (SNP) analysis supports a reevaluation of the phylogenetic relationships between the classical *Bordetella* subspecies, and suggests a closer link between ovine and human *B. parapertussis* lineages than has been previously proposed. Comparative analyses of genome content revealed that only 50% of the pan-genome is conserved in all strains, reflecting substantial diversity of genome content in these closely related pathogens that may relate to their different host ranges, virulence and persistence characteristics. Strikingly, these analyses suggest possible horizontal gene transfer (HGT) events in multiple loci encoding virulence factors, including O-antigen and pertussis toxin (Ptx). Segments of the pertussis toxin locus (*ptx*) and its secretion system locus (*ptl*) appear to have been acquired by the classical *Bordetella* subspecies and are divergent in different lineages, suggesting functional divergence in the classical Bordetellae.

**Conclusions:**

Together, these observations, especially in key virulence factors, reveal that multiple mechanisms, such as point mutations, gain or loss of genes, as well as HGTs, contribute to the substantial phenotypic diversity of these versatile subspecies in various hosts.

## Background

The genus *Bordetella* contains nine designated species, three of which are so closely related that they are considered subspecies and are referred to as the “classical *Bordetella*”; *B. pertussis, B. parapertussis* and *B. bronchiseptica*[1]. These three present an outstanding experimental system because, although they are highly similar at the DNA sequence level, they vary in critical aspects of bacterial pathogenesis, including host specificity, severity of diseases, and duration of infections (acute versus chronic) [2]. For example, *B. bronchiseptica* causes infections ranging from lethal pneumonia to asymptomatic respiratory carriage [2] and chronically colonizes the respiratory tracts of various mammalian hosts, with some lineages primarily isolated from humans [3]. *B. pertussis* and *B. parapertussis*_*hu*_*,* which are thought to have evolved independently from a *B. bronchiseptica*-like progenitor [4], are causative agents of whooping cough in humans. Another distinct lineage only isolated from sheep has been designated *B. parapertussis*_*ov*_[5]. The very close evolutionary relationship between these subspecies and the diversity of their interactions with various hosts provides an opportunity to explore the evolutionary genetic basis for changes in important pathogen characteristics.

The prior description of a single genome of each of the classical *Bordetella* subspecies reported an extensive number of gene inactivations, deletions and genomic rearrangements, as associated with host-adaptation of other pathogens, such as *Yersinia pestis*[2]. However, unlike *Y. pestis* and *E. coli,* there is limited evidence of gene acquisition amongst the *Bordetella* species. Microarray-based comparative genome hybridization (CGH) has suggested that the more distantly related *B. holmesii* may have obtained some genes from *B. pertussis*, but did not reveal evidence of gene acquisition or exchange within the classical Bordetellae [6]. We have previously reported evidence that the distributions of subsets of the O-antigen encoding genes correlate with O-antigen type, but not with MLST-based phylogeny of *B. bronchiseptica* strains [7]. However, genomic-signatures associated with horizontal gene transfer (HGT) events have not been described for the classical Bordetellae. Furthermore, the genomic plasticity of these pathogens, which is related to novel gene acquisition [8], has not been investigated. Understanding the pan-genome of the classical Bordetellae and identifying genes that may have been acquired at important points in their natural evolutionary history will provide insight into the potential role of HGTs in their past and ongoing evolution.

In this study, we sequenced seven divergent classical *Bordetella* isolates, including five *B. bronchiseptica* complex I (non-human) and IV (human) strains, one *B. parapertussis*_*ov*_ (sheep) strain, and one *B. pertussis* (human) strain. Phylogenetic trees of the classical Bordetellae based on genome-wide single nucleotide polymorphism (SNP) data provide a more detailed and robust view of their genetic relationship, as well as some new insights. For example, a shared evolutionary history for *B. pertussis* and *B. bronchiseptica* complex IV strains, and a closer relationship between *B. parapertussis* isolates of ovine and human sources. Genome content analyses revealed that only approximately 50% of genes are shared by all strains in the core genome consisting of 2,857 gene families. Variably present genes encode a range of functions, including many potential virulence factors, in a pan-genome of at least 5,558 gene families. Notably, mathematical analysis of these genomes suggests that the classical Bordetellae pan-genome is “open” with limited gene acquisition. Additional evidence of HGT events are identified within the loci of genes required for Pertussis Toxin (Ptx) assembly and secretion, suggesting that HGT, followed by divergent evolution, contribute to strain diversity and host adaptation of the classical *Bordetella* subspecies.

## Results

### Sequencing of seven new classical *Bordetella* genomes

In 2003, Parkhill *et al.* published the first comparative genomic analysis of *B. pertussis*, *B. parapertussis*, and *B. bronchiseptica*[2]. Since then, *B. petrii*[9], *B. avium*[3], and seven *B. pertussis* strains [10,11] have been sequenced. Here, we sequenced seven divergent classical *Bordetella* strains, including five *B. bronchiseptica* strains isolated from both human and non-human hosts (strains 253, 1289, MO149, Bbr77, and D445), one *B. parapertussis ovine* strain (Bpp5), and one *B. pertussis* strain (18323) to comprehensively examine the evolutionary relationships of the classical Bordetellae (Table 1).

**Table 1:** Summary of sequenced strain information

**Strain**	**ST**	**Species**	**Host**	**Location**	**Year**	**Complex**	**Reference**	**Note**
**RB50**	12	*B. bronchiseptica*	Rabbit	USA	Unknown	I	[2]	Published
**Tohama I**	1	*B. pertussis*	Human	Japan	1954	II	[2]	Published
**CS**	1	*B. pertussis*	Human	China	1951	II	[11]	Published
**B0558**	2	*B. pertussis*	Human	Netherlands	1949	II	[10]	Published
**B1193**	1	*B. pertussis*	Human	Netherlands	1950	II	[10]	Published
**B1831**	2	*B. pertussis*	Human	Netherlands	1999	II	[10]	Published
**B1834**	2	*B. pertussis*	Human	Netherlands	1999	II	[10]	Published
**B1917**	2	*B. pertussis*	Human	Netherlands	2000	II	[10]	Published
**B1920**	2	*B. pertussis*	Human	Netherlands	2000	II	[10]	Published
**12822**	19	*B. parapertussis*_*hu*_	Human	Germany	Unknown	III	[2]	Published
**1289**	32	*B. bronchiseptica*	Monkey	South America	Unknown	I	[12]	This study
**253**	27	*B. bronchiseptica*	Dog	USA	Unknown	I	[13]	This study
**MO149**	15	*B. bronchiseptica*	Human	USA	Unknown	IV	[4]	This study
**D445**	17	*B. bronchiseptica*	Human	USA	Unknown	IV	[4]	This study
**Bbr77**	18	*B. bronchiseptica*	Human	Germany	Unknown	IV	[4]	This study
**18323**	24	*B. pertussis*	Human	USA	1946	II	[14]	This study
**Bpp5**	16	*B. parapertussis*_*ov*_	Sheep	New Zealand	Unknown	I	[15]	This study

Strain name, sequence type based on MLST (ST), species, host, isolation location, isolation year, complex based on MLST [4], and references for each strain are summarized.

*B. bronchiseptica* strain 1289 was sequenced and assembled at the Pennsylvania State University using Illumina [16], 454 with GSFLX Titanium chemistry [17,18], and optical maps [19] from OpGen (Gaithersburg, MD). The other six strains were sequenced at the Sanger Institute. The genomes of Bpp5, 253, MO149, and 18323 were sequenced to approximately 6 to 9-fold coverage with ABI3730 sequencers [20]. The genomes of D445 and Bbr77 were sequenced on Illumina [16] and 454 [17,18]. Read assembly resulted in finished genomes of MO149, Bpp5, 1289 and 18323, 4 contigs in 253, 11 scaffolds in D445, and 16 scaffolds in Bbr77. The sum of the contig or scaffold lengths ranged from 4.1 Mb to 5.2 Mb, which is comparable to genome sizes of previously sequenced strains.

These new genomes and previously sequenced genomes share similar characteristics, such as G + C content, number of rRNA operons, or tRNAs (Table 2). The *B. bronchiseptica* strains have the largest genomes (~5.3 MB), followed by *B. parapertussis* strains (~4.8 MB) and *B. pertussis* strains (~4.1 MB), as previously observed [2]. This work confirmed the presence of different Insertion Sequence (IS) elements in different species [5]. Overall, even though these strains have shown diverse phenotypes in the mouse model of infection [2,4,12-15] and were isolated from different hosts, they appear to share similar genomic organization and characteristics.

**Table 2:** Summary of sequenced genome annotation information

**Species**	***B. bronchiseptica***						***B. parapertussis***			***B. pertussis***		
**Strain Name**	RB50	1289	253	MO149	Bbr77	D445	12822	Bpp5	Bpp5 plasmid	Tohama I	18323	CS
**Contig (C) / Scaffold (S)**	1	1	4 C	1	16 S	11 S	1	1	1	1	1	1
**Genome Size (bp)**	5339179	5208522	5264383	5091817	5115717	5243194	4773551	4887379	12195	4086189	4043846	4124236
**Gaps (Ns)**	0	628	2	0	146101	251924	0	0	0	0	0	0
**G + C content (%)**	68.49	68.65	68.64	68.86	68.51	68.23	68.43	68.15	61.39	68.12	68.11	68.1
**Predicted CDSs**	5009	4785	4845	4669	4667	4775	4402	4558	15	3806	3746	3822
**Pseudogenes**	12	39	60	44	76	256	217	389	0	359	369	357
**CDSs with Ns**	0	0	0	0	320	473	0	0	0	0	0	0
**Average Gene Size (bp)**	983	1000	989	1002	974	953	1000	986	534	983	986	991
**% of CDSs**	92.23	91.9	91.04	91.88	91.51	91.21	92.23	92.16	65.6	91.62	91.38	91.84
**rRNA operons**	3	3	3	3	3	3	3	3	0	3	3	3
**tRNA**	55	54	54	54	54	54	54	54	0	51	51	51
**IS481**	0	0	0	0	0	0	0	0	0	230	239	236
**IS1001**	0	0	0	0	0	0	22	27	0	0	0	0
**IS1002**	0	0	0	0	0	0	9	0	0	5	7	4
**IS1663**	0	0	0	7	0	9	0	0	0	17	18	18

Strain name, number of contigs or scaffolds, genome size (bp), gaps (Ns), G + C content (%), number of predicted coding sequences (CDSs), number of pseudogenes, number of CDSs with Ns due to sequence gap, average gene size (bp), percentage of all coding sequences, number of rRNA operons, number of tRNAs, and number of each IS elements are summarized.

**Figure 1:**
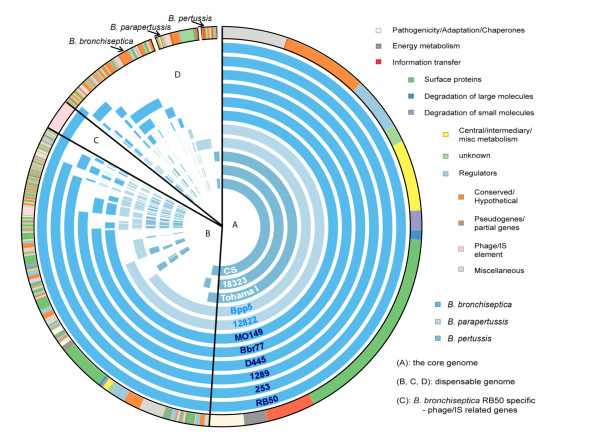
**Comparative genome content of eleven classical Bordetellae strains.** The outermost circle indicates the predicted functional categories of each gene families in the pan-genome. Species-specific gene families are individually blocked with black line (*B. bronchiseptica, B. parapertussis*, and *B. pertussis*). Internal circles indicate the presence (solid color) or absence (unfilled) of each gene family in each strain examined. Circles from outer to inner are started with *B. bronchiseptica* strains followed by *B. parapertussis* strains and *B. pertussis* strains. Gene families that are shared in all strains are in the region **A**, while gene families that are not conserved in all strains are in the region **B**, **C**, and **D**. Region **C** indicates the phage encoding gene families that are only present in RB50. This figure was created using the Circos software [21].

*B. pertussis* strains (Tohama I, 18323, and CS) contained no novel genes except transposases or insertion elements, confirming that *B. pertussis* has evolved from a *B. bronchiseptica*-like ancestor by genome decay (Figure 1). While we did not observe many genome rearrangements between Tohama I and CS, extensive genome rearrangement distinguishes Tohama I and 18323 (*unpublished*). More phage-related and membrane protein encoding genes are present in *B. bronchiseptica* non-human isolates (RB50, 253, and 1289), while more transposases are present in *B. bronchiseptica* human-isolated strains (D445, Bbr77, and MO149). Notably, a locus novel to the classical Bordetellae was observed in both D445 (D445_1578 – D445_1707) and Bbr77 (Bb77_4113 - Bbr77_4216) strains. This locus is also present in *B. petrii* and includes a Type IV secretion system-like locus, which is being further investigated (*unpublished*). *B. parapertussis*_*ov*_ Bpp5 strain has about 150 strain-specific gene families comprised mostly of hypothetical proteins, phage-related proteins, and putative secreted proteins. Comparing the two lineages designated *B. parapertussis*, there are about 300 gene families that are present in either *B. parapertussis*_*hu*_ or *B. parapertussis*_*ov*_, but not both. Most of these gene families are shared with *B. bronchiseptica* strain RB50 and include exported proteins, hypothetical proteins, and membrane proteins. While *B. parapertussis*_*hu*_ 12822 is missing the locus encoding a putative type II secretion system, *B. parapertussis*_*ov*_ strain Bpp5 is missing the locus encoding flagella. Furthermore, Bpp5 has a unique 12 KB plasmid containing genes predicted to encode proteins involved in partition, replication, plasmid conjugal transfer, mobilization and transcriptional regulation. We have not observed this plasmid in any other classical *Bordetella* strains.

### The classical *Bordetella* pan-genome and core genome

**Figure 2:**
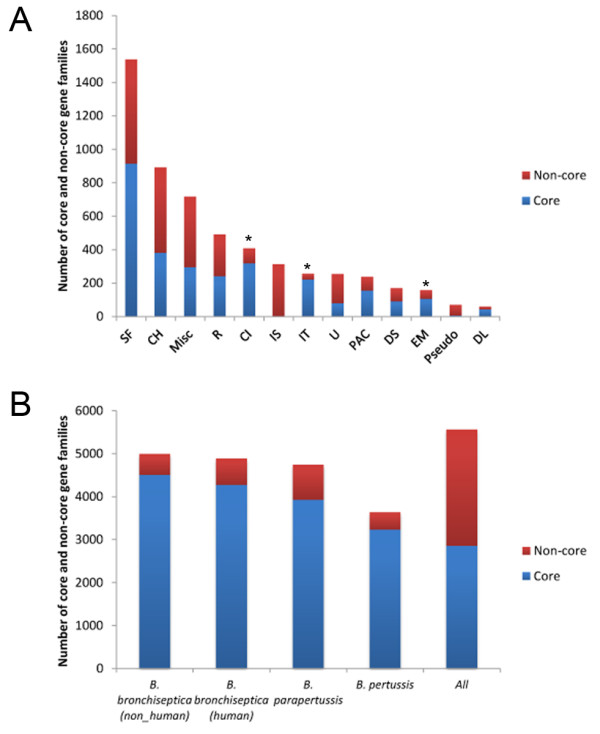
**Core and non-core genome of eleven classical Bordetellae pan-genome.** The number of core and non-core gene families in each predicted functional category of the classical Bordetellae pan-genome (**A**) and each species pan-genome (**B**) are summarized (*B. bronchiseptica* non-human isolates: RB50, 253 and 1289, *B. bronchiseptica* human isolates: MO149, D445, and Bbr77, *B. parapertussis* strains: 12822 and Bpp5, and *B. pertussis* strains: Tohama I, CS and 18323). Functional categories are **SF:** surface proteins, **CH:** conserved hypothetical proteins, **Misc:** miscellaneous information, **R:** regulators, **CI:** central/intermediary metabolism, **IS:** phage/insertion sequence (IS) elements, **IT:** information transfer proteins, **U:** unknown proteins, **PAC:** pathogenicity/adaptation/chaperones, **DS:** degradation of small molecules, **EM:** energy metabolism, **Pseudo:** pseudogenes and **DL:** degradation of large molecules. * indicates the gene families that more than 80% of gene families are conserved in all strains.

To determine the global gene repertoire (pan-genome) and the universally shared “core” genome, we identified orthologous gene families via OrthoMCL [22,23] using all of the published *Bordetella* genomes that have manually curated annotations (RB50, Tohama I, CS, 12822, *B. petrii*, and *B. avium*). The pan-genome of these strains has 8,425 gene families, consisting of 1,778 core gene families and 6,647 gene families that are missing in at least one strain (see Additional file 1). While four *Bordetella* species remain to be sequenced and others are not finished or adequately annotated, these results do provide solid, yet preliminary, insight into the global gene repertoire of *Bordetella*. Throughout the remainder of this study, all investigations will focus on the evolutionary relationship of the classical Bordetellae. The classical *Bordetella* pan-genome includes 5,558 gene families, consisting of 2,857 core gene families (Region A in Figure 1) and 2,701 non-core gene families (Regions B, C and D in Figure 1) (see Additional file 2). The core gene families are mainly involved in energy metabolism, central metabolism and information transfer (Figure 2A). The most abundant gene family groups in these genomes encode surface proteins and 40% of these belong to the non-core genome. Variability in these proteins, which are likely surface exposed and antigenic, would be expected to affect host immune recognition [24].

When bacteria specialize to an ecological niche, this adaptation is often accompanied by a loss of genes that are no longer required or beneficial in the new niche [25]. This concept can explain the large-scale genome loss exhibited by *B. pertussis* and *B. parapertussis*, which appear to have evolved from a zoonotic generalist into specialized human pathogens [2]. If other lineages had adapted to particular environments (i.e. subsets of hosts), we would expect those lineages to lose genes not required in that environment, but retain a set of shared core genes required for their shared characteristics, such as the ability to infect the respiratory tract of their particular host. *B. bronchiseptica* complex I non-human isolates (RB50, 253, and 1289; sharing 4,509 gene families) and complex IV human isolates (MO149, Bbr77 and D445; sharing 4,271 gene families) strains have larger core genomes, than *B. pertussis* strains (Tohama I, 18323, and CS; sharing 3,235 gene families) (Figure 2B) [4]. In fact, the core genome of all available classical *Bordetella* strains excluding *B. pertussis* isolates (3,652 gene families) was larger than that of *B. pertussis* strains, suggesting that this lineage has selectively lost genes/functions that are retained in all others. The non-core genome (2,701 gene families not present in all strains) contains a large variety of gene families and many phage-encoding genes, and likely contributes to the phenotypic variation of this group.

### Open pan-genome with limited gene acquisition

**Figure 3:**
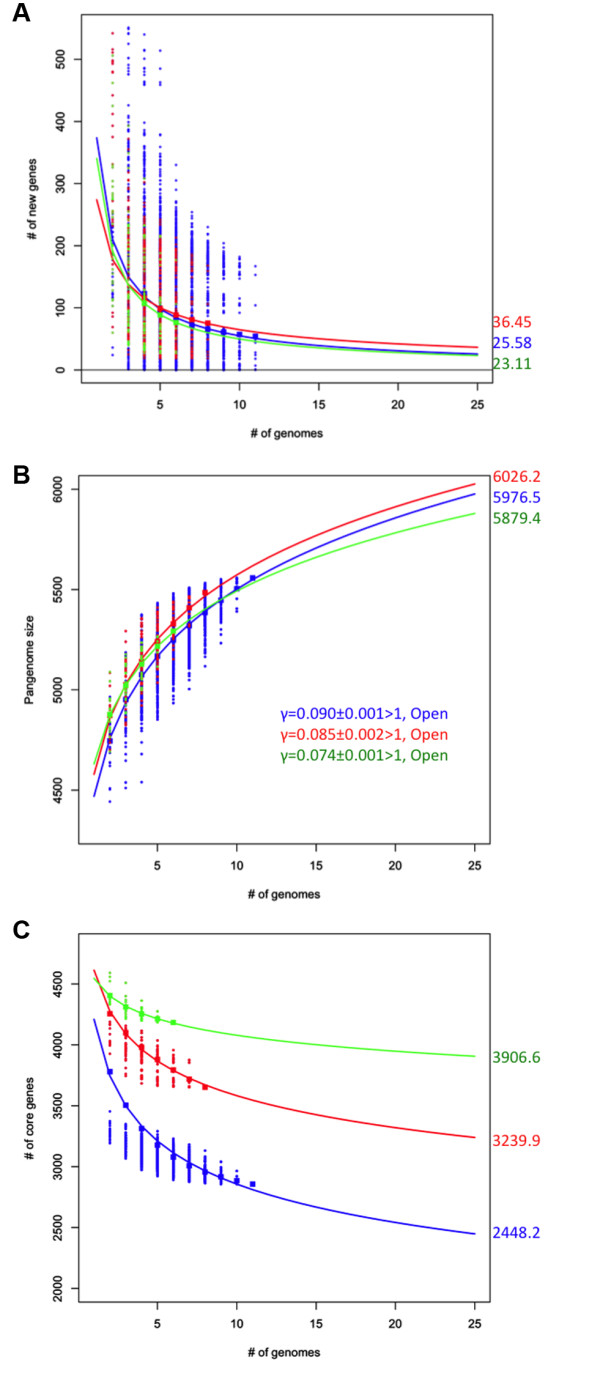
**Mathematical estimation of size of novel gene families, pan-genome, and core gene families.** The number of novel gene families (**A**), pan-genome size (**B**), and the number of core gene families (**C**) were estimated for the classical Bordetellae (blue: all eleven genomes), *B. bronchiseptica* strains only (green: RB50, 253, 1289, MO149, D445, and Bbr77), and all the strains except *B. pertussis* strains (red: RB50, 253, 1289, MO149, D445, Bbr77, 12822, and Bpp5). If n genomes are selected from 11, there are 11!/ [(n-1)!*(11-n)!] possible combinations. Each possible combination is plotted as a point, and the line is fitted to the power law model adapted from the methods of Tetellin *et al*. [26]. γ in the power law model for the pan-genome size estimation was reported for each group. The numbers shown on the right side of each graph are the number of expected novel gene families, pan-genome size, and core gene families with 25 genomes.

To evaluate and compare the total gene pool of all classical *Bordetella* subspecies (i.e. all eleven sequenced strains), compared to that of all strains excluding *B. pertussis* strains, and that of *B. bronchiseptica* strains alone (RB50, 253, 1289, MO149, Bbr77, and D445), we employed the prediction method described by Tettelin *et al.*[26]. Based on the number of novel gene families per additional genome analyzed, all of the groups (the classical *Bordetella* subspecies, all the strains excluding *B. pertussis* strains, and *B. bronchiseptica* strains only) appeared to have what Tettlin *et al.* described as open but “slowly closing” pan-genomes [27] (Figure 3A). This indicates that more sequenced genomes have the potential to provide additional genomic diversity but that beyond a couple of hundred sequenced genomes novel genes are less likely to be discovered.

A complementary approach to assess gene acquisition was used to calculate the increase in pan-genome size with each additional genome sequenced (Figure 3B). This expected pan-genome size (*n*) upon sequencing an *Nth* genome was modeled by the power law function *n* = *κN*^*γ*^, where an open pan-genome has a γ value greater than zero and less than one, with lower values signifying a more closed genome with fewer acquired genes. The γ value for the classical *Bordetella* subspecies (0.090) is lower than that for *Bacillus cereus* (0.43) [28], indicating that the pan-genome of the classical Bordetellae is open but grows more slowly than that of *B. cereus* with each additional genome sequenced. With 25 additional genomes, the pan-genome of the classical *Bordetella* subspecies will reach approximately 6,000 gene families, while the pan-genome of the group excluding *B. pertussis* strains is estimated to contain 6,026 gene families. Furthermore, since a smaller core genome size reflects smaller sets of shared functional genes, the small size of the core genome containing *B. pertussis* strains is additional evidence that this lineage has lost the functions encoded by approximately 800 genes (Figure 3C). This further supports the previous hypothesis that *B. pertussis* strains have adapted to their niche by genome reduction [2] and that there is a correlation between the pan/core genome size and the host range.

### Variations in virulence factors amongst the classical Bordetellae

**Figure 4:**
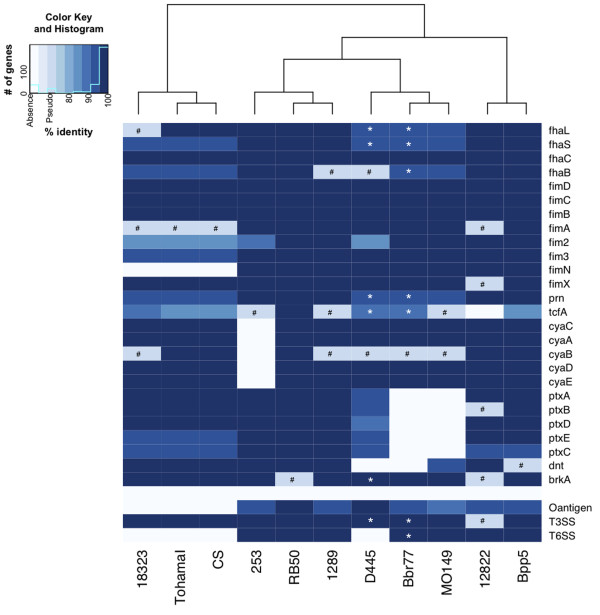
**Diversity in virulence factor genes/loci among the classical Bordetellae compared to RB50.** The heatmap was generated based on nucleotide percentage identity compared to RB50 for each gene/loci. Absence of a certain gene and presence of a pseudogene are highlighted with white and sky blue color with #, respectively. * indicates the missing nucleotides due to the draft status of the genome. The gene content tree, the dendrogram of hierarchical clustering of the complete pan-genome matrix, was superimposed on top of the heatmap. Manhattan distances, linkage method, and 1,000 bootstrap replicates were used for the clustering.

To examine the possible basis for differences in virulence phenotypes amongst strains/lineages, we compared the presence/absence of factors thought to be involved in interactions with the host, loosely referred to as “virulence factors”, including filamentous hemagglutinin (FHA), fimbriae (Fims), pertactin (PRN), tracheal colonization factor (TcfA), adenylate cyclase/hemolysin (ACT), dermonecrotic toxin (Dnt), pertussis toxin (Ptx), *Bordetella* resistance to killing (BrkA) protein [29], O-antigen [30], Type III secretion system (TTSS) [31], and Type VI secretion system (T6SS) [32]. Only genes encoding FHA, PRN, and TTSS locus are conserved in all strains (Figure 4). *B. bronchiseptica* complex IV strains (D445, Bbr77, and MO149) have the most divergent *fha* locus (as low as 92% identity) compared to RB50, while *B. pertussis* strains have the most divergent *fim* loci (as low as 84% identity) with gene deletions and multiple internal sequence variations. Although *tcfA* varies in all strains (as low as 84% identity), genes encoding PRN segregate into clear distinctive groups between *B. bronchiseptica* complex I/*B. parapertussis* strains and *B. bronchiseptica* complex IV/*B. pertussis* strains, as previously described [4]. Together, the presence, absence or divergence of these virulence factors do not always follow the phylogenetic relationships, suggesting some other processes, in addition to sequence divergence by accumulation of point mutations, may contribute to variation of virulence factor genes.

In a prior analysis of the hypovirulent *B. bronchiseptica* strain 253, we observed that it does not contain the whole ACT locus [13]. Notably, strains 1289, D445, Bbr77, MO149, and 18323 all contain a frameshift mutation within *cyaB*; however, each strain remains beta-hemolytic and grows within the murine respiratory tracts (*unpublished*). Therefore, the functional effects of this mutation on ACT secretion and hemolysis appear less dramatic than that of the mutation in strain 253. Strikingly, strains Bbr77 and MO149 do not contain the entire *ptx* locus and the other human isolate (D445) has a divergent *ptx* locus (as low as 88% identity) compared to RB50. *dnt* is divergent (as low as 93% identity) or missing in complex IV strains compared to RB50. One of the complement resistance factors, *brkA,* is very similar (>97% identity) in all strains except that it is a pseudogene in 12822 and RB50. Combined, these data reveal a discord between the phylogenetic relatedness of strains and the variation in their individual virulence genes.

In addition to small loci (<10 kb) responsible for encoding adhesins and toxins, we compared large loci (>10 kb) containing contiguous genes with highly coordinated functions. The T3SS locus is very similar (>98% identity) among all strains, with the notable exception being a pseudogene present only in *B. parapertussis*_*hu*_ strain 12822 [12,33]. However, other loci were much more variable. Consistent with previous reports [2,4,7], the genetic makeup of the O-antigen locus (0-26 kb) widely fluctuated among strains, with the entire locus missing in all three *B. pertussis* stains. In strains in which they are present, these genes share as little as 90% identity with their apparent orthologs. In some cases, the locus includes completely different sets of genes. Two pseudogenes are present within the O-antigen locus in Bpp5, suggesting relatively recent loss of function. Similarly, a locus encoding a putative T6SS shows a high degree of variations. For example, *B. pertussis* strains and D445 are missing parts of the T6SS locus, while both human and ovine *B. parapertussis* strains either have pseudogenes or are missing subsets of genes within this locus. A predicted pseudogene is also present in this locus of *B. bronchiseptica* strain 1289. Together, these data reveal the intriguing tendency of virulence loci to be lost or divergent in the human isolates (*B. bronchiseptica* complex IV strains, *B. parapertussis*_*hu*_ strains*,* and *B. pertussis* strains), possibly signifying differential roles in different hosts [34].

### Phylogenomic analysis

The observation that there are discrepancies between the phylogenetic relationships of sets of genes suggests that a small set of genes may be limited in its ability to accurately represent phylogenetic relationships among these strains [35]. Thus, to more accurately and robustly evaluate the genetic relationships between *Bordetella* isolates, we built a phylogenetic tree with all the genomic content, using a previously defined pan-genome matrix in our analysis. This matrix was constructed with genomes and gene families as columns and rows, respectively. Each cell in the matrix presents 1 or 0, depending on the presence or absence of the gene family in each genome. By generating a gene content tree based on this matrix, excluding unique gene families in each strain, we observed many similarities and some notable differences as compared to earlier studies that only used a small set of housekeeping genes. *B. pertussis* strains form a separate lineage that is isolated from the other two species (Figure 4). This clustering trend can be explained by the drastic genome reduction (absence of genes) in *B. pertussis* strains. However, *B. parapertussis* strains are closely related to *B. bronchiseptica* strains*,* reflecting a relatively large set of shared core genes. Notably, the *B. parapertussis*_*ov*_ strain (Bpp5) clustered together with *B. parapertussis*_*hu*_ strain 12822, consistent with the species designation but different from the previous minimum spanning tree based on MLST [4], which clustered Bpp5 within *B. bronchiseptica* complex I strains and separated 12822 from *B. bronchiseptica* strains.

**Figure 5:**
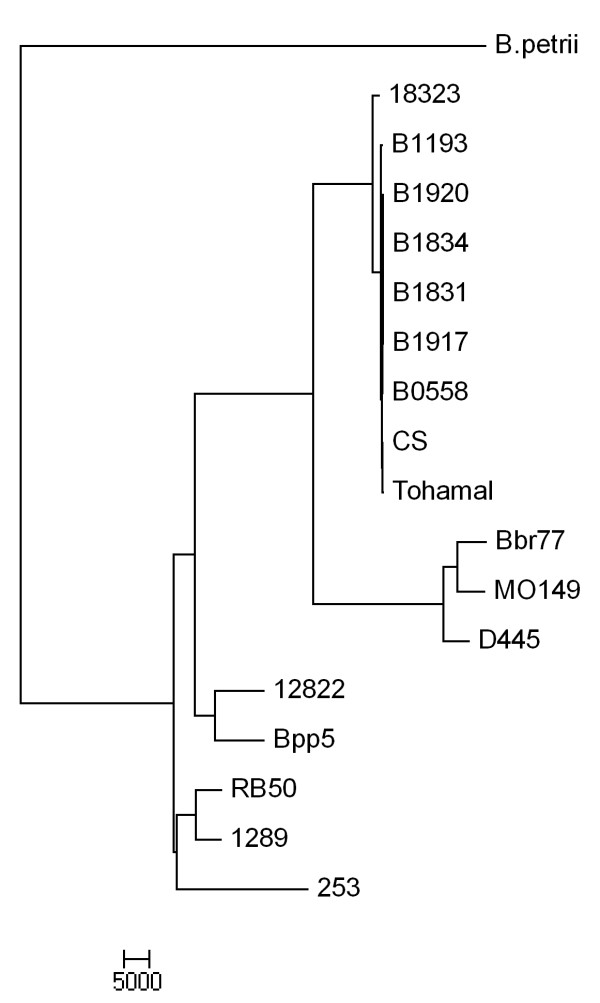
**Maximum likelihood phylogenetic tree of eleven classical Bordetellae with genome-wide SNP sites.** The phylogenetic tree was reconstructed with genome-wide SNP sites based on *B. bronchiseptica* RB50.

To include all the additional information contained in the individual SNPs, a phylogenetic tree was generated based on genome-wide SNP candidate sites against the reference genome (RB50) with *B. petrii* as an outgroup (Figure 5). Similar to the previously published MLST minimum spanning tree, this SNP-based tree suggests that *B. pertussis* is closely related to *B. bronchiseptica* complex IV strains [4]. Moreover, building the phylogenetic tree using genome-wide SNPs supports two important genetic relationships that previous phylogenetic analyses did not reveal. First, *B. pertussis* and *B. bronchiseptica* complex IV appear to share a more recent common ancestor distinct from that shared by *B. parapertussis* and *B. bronchiseptica* complex I strains. Second, *B. parapertussis*_*ov*_ strain Bpp5 is more closely related to *B. parapertussis*_*hu*_ strain 12822 than to *B. bronchiseptica* complex I strains. The agreement on the closer relationship between the two *B. parapertussis* clades of both the SNP-based and gene content-based trees suggests that the more distant relationship observed in the previously published MLST-based tree is likely due to the limited amount of information in the seven housekeeping genes that were used.

SNP densities across each genome, compared to the RB50 genome, differ between strains. *B. bronchiseptica* complex IV strains (MO149, D445, and Bbr77) have the highest overall density of SNP sites (~12 SNPs/1,000 bp), followed by *B. pertussis* strains (~9 SNPs/1,000 bp), *B. bronchiseptica* complex I strain 253 (~7 SNPs/1,000 bp), *B. parapertussis* strains (~4 SNPs/1,000 bp), and complex I strain 1289 (~2 SNPs/1,000 bp), reflecting their overall relatedness to RB50. Of all SNPs, about 61% are synonymous (average dS = 0.0170), while ~26% are non-synonymous (average dN = 0.0023), reflecting the overall pressure of purifying selection. ~12% of SNPs were found within intergenic regions, and approximately 1% are in pseudogenes. Genes encoding phage-related, hypothetical proteins and some virulence factors, including *ptx,* contained amongst the highest SNP densities (see Additional file 3). One explanation for this observation could be positive selection, for example via the proposed vaccine-driven selection for antigenic variation of genes encoding vaccine components [37,38]. However, dN/dS ratios are lower than one in most of these genes, indicating overall negative (purifying) rather than positive (diversifying) selection. These results do not support the view that the normal rate of SNP generation, followed by positive selection for variation, resulted in this diversity, and raise the possibility that other mechanisms of diversity generation, such as insertions or deletions, HGT and functional divergence, occurred within these discrete loci.

### Horizontal gene transfer and divergent evolution of the classical Bordetellae

**Figure 6:**
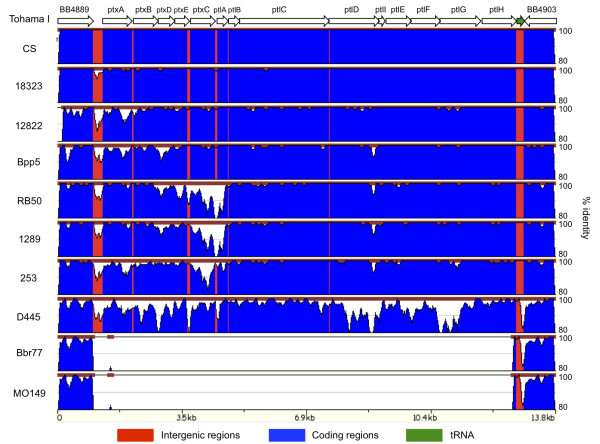
**Percent sequence similarity of *ptx/ptl *locus with flanking genes against Tohama I.** Percent sequence similarity of the *ptx/ptl* locus and flanking genes based on Tohama I was plotted between 80% and 100% using zPicture [36]. Intergenic regions, coding regions, and a tRNA were highlighted with red, blue, and green, respectively.

To more closely examine the divergence of the *ptx/ptl* loci, we compared the percent sequence similarity of these genes and their flanking genes in all sequenced strains to that of Tohama I (Figure 6). The *ptx/ptl* loci of *B. pertussis* 18323 and CS and *B. parapertussis* 12822 and Bpp5 are closely related to that of Tohama I (>98% and >95% identity, respectively), while those of *B. bronchiseptica* non-human strains are much more divergent (as low as 87% identity). Conversely, *B. bronchiseptica* human isolates, which are much more closely related based on overall SNPs (Figure 5), have either low percentage similarity to Tohama I (D445; as low as 82% identity) or do not contain the entire *ptx/ptl* loci (Bbr77 and MO149) (Figure 6). There were ten or less SNPs distinguishing the *ptx/ptl* loci of seven recently published *B. pertussis* genomes (see Additional file 4). The relatively high SNP densities of the *ptx*/*ptl* loci do not appear to be due to positive selection because pair-wise dN/dS ratios are below one, indicating overall purifying selection (see Additional file 5).

An alternative explanation for the high SNP density in the *ptx/ptl* loci could be the introduction of variation via horizontal gene transfer (HGT). Gerlach *et al.* previously speculated that the *ptx/ptl* loci had features of pathogenicity islands (PAIs) based only on the clusters of virulence genes in these loci, the presence of a tRNA (known to be associated with HGT and often a DNA integration target site [39]), and the absence of these loci in some strains [40]. Using the entire sequences of all eleven classical *Bordetella* strains, we analyzed each genome with Alien_hunter [41], which detects horizontally transferred genome segments. Nine to eighteen percent of each genome was identified as containing potential HGT candidates based on atypical genomic composition (see Additional file 6). The loci encoding phage-related proteins, alcaligin biosynthesis proteins, cytochrome ubiquinol oxidase, NADH-ubiquinone oxidoreductase, ATP synthase and many adhesins were identified as HGT candidates. Among the candidates were a number of genes required for assembly of O-antigen, consistent with our prior prediction of HGT within this locus [7,42,43]. Notably, Alien_hunter also predicted that the *ptx* locus and a part of its associated secretion system (*ptl*) locus were acquired by HGT.

**Figure 7:**
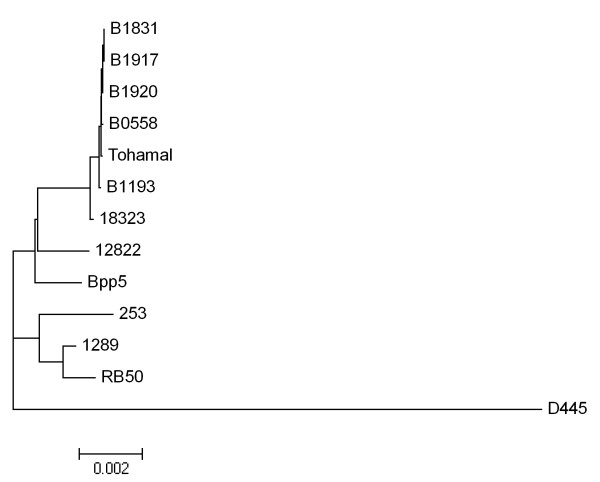
**The phylogenetic tree of the *ptx/ptl *locus.** The tree was reconstructed by maximum likelihood methods with 1,000 bootstrap replicates based on the *ptx/ptl* locus sequences. B1920 represents B1834 and Tohama I represents CS because they have the identical sequences.

To further investigate possible HGT of *ptx/ptl* genes, phylogenetic trees were generated based on individual genes within the *ptx/ptl* loci; these gene trees (Figure 7 and see Additional file 7) were then compared to the genome-wide SNP tree (Figure 5) to identify incongruence in the tree topology. The majority of the *ptx/ptl* gene trees are similar to the genome-wide SNP tree in that each strain is clustered based on their species designation, such as *B. pertussis*, *B. parapertussis*, and *B. bronchiseptica* non-human strains. However, the *B. bronchiseptica* human isolate D445 locus is located on a long branch separate from all the other strains, implying that the *ptx/ptl* loci in D445 has an evolutionary history different from other *Bordetella* species. Additionally, *B. pertussis* clades and *B. parapertussis* clades clustered together in the individual gene trees unlike in the genome-wide SNP tree. These results suggest HGT being a source of diversity within this locus, although they do not rule out other possibilities.

In addition to the higher overall SNP density, the distribution of SNPs across the *ptx/ptl* loci appears to be non-random, with areas of low density (consistent with the flanking genes and much of the genome) and areas of much higher density. For example, *B. bronchiseptica* complex IV strains have accumulated the highest density of SNPs (D445) or have lost the entire locus (MO149 and Bbr77), suggesting a change in the requirement for Ptx in this lineage. However, much of the *ptl* locus of the non-human *B. bronchiseptica* strains (RB50, 1289, and 253) have low SNP density, while the *ptx* locus, most notably the *ptxC* gene, has much higher SNP density (Figure 6). Interestingly, the large majority of these SNPs across the locus result in silent (synonymous) mutations (dN/dS < 0.6), suggesting the high SNP density is not due to positive selection for variations. It is also interesting to note that sequence similarity differs between the *ptx* locus (average 96% identity) and the *ptl* locus (average 98% identity) in all the strains except strain D445, suggesting a different evolutionary history for the *ptx* and *ptl* loci. Together, the *ptx/ptl* loci appear to have been horizontally transferred to the classical Bordetellae and have diverged in different lineages.

## Discussion

The recent availability of classical *Bordetella* genomes gives us insight into the evolutionary changes involved in their divergence, including extensive genome decay, rearrangements and, for the first time, specific sequence data revealing evidence of limited gene acquisition [2,5]. Our mathematical prediction suggests that we will need over a hundred genomes to comprehensively describe a classical *Bordetella* pan-genome, although these numbers may change when more strains are added to this analysis (Figure 3). The core genome of the classical Bordetellae represents only a small fraction (~50%) of the pan-genome, and many genes in the non-core genome are likely to contribute to the diverse characteristics of these strains (Figures 1 and 2). Substantial variation is observed in virulence factor genes in particular, with human isolates appearing to have more inactivated genes than non-human isolates (Figure 4). Some of these changes are similar in strains with a shared evolutionary history, reflected by the genome-wide SNP-based phylogenetic tree. This tree is similar to the previously constructed MLST minimum spanning tree [4] with two important differences. The first is that the branching pattern of the genome-wide SNP tree links human and ovine strains of *B. parapertussis*, which can explain their shared characteristics that led to their similar species designation. This common branch suggests some divergence from *B. bronchiseptica* complex I prior to the divergence of these two lineages. Second, a more recent last common ancestor was shared by *B. pertussis* and *B. bronchiseptica* complex IV, making them more closely related to each other than to the other lineages (Figures 5). The resolution of our genome-wide SNP tree also provides a firm foundation for the comparisons with trees built based on individual genes, supporting or refuting the possibility of HGT candidates identified by Alien_hunter (Figures 6 and 7).

Species with limited diversity, such as *Chlamydia trachomatis* and *Bacillus anthracis,* share ~90% of their closed pan-genomes [44,45], while species with open pan-genomes, such as *E. coli,* can share as little as 20% of their pan-genomes, with nearly 80 new genes discovered for each additional sequenced genome [46]. Like *Streptococcus pneumoniae*, *B. bronchiseptica* has an open pan-genome, but the number of novel gene families is likely to decrease more rapidly with additional genomes sequenced, suggesting an intermediate category between open and closed pan-genome (Figure 3) [27]. Pan-genome size often reflects host range and gene pool availabilities. For example, a human pathogen restricted to human oral-nasal mucosa, *Streptococcus pyogenes,* has a smaller pan-genome than that of a zoonotic pathogen capable of infecting multiple organs, *Streptococcus agalactiae*[47]. Although we could not use our computational analysis to estimate the pan-genome sizes of *B. parapertussis* and *B. pertussis* due to limited number of fully annotated genomes (Figure 3), we speculate that the pan-genome of these two species would be smaller than that of *B. bronchiseptica* due to restricted host range, consistent with our core and pan-genome analysis using eleven genomes (Figure 2). This suggests that the larger pan-genome of *B. bronchiseptica* may be essential for survival in a broader niche of multiple host species (Figures 1 and 2). Furthermore, a similar analysis comparing two *B. bronchiseptica* complexes (I versus IV) with more strains may elucidate how some *B. bronchiseptica* isolates that are more closely related to *B. pertussis* share the propensity for isolation from human hosts.

Many known virulence factor genes or loci are not conserved in all the classical *Bordetella* strains, and the presence, absence, or inactivation of these virulence factors does not always follow the phylogenetic species relationships (Figures 4 and 5). Our study suggests that limited HGTs between foreign species and the classical Bordetellae have occurred, resulting in an open pan-genome with limited growth. Although we cannot determine the precise origin of horizontally transferred loci due to limited availability of other closely related *Bordetella* genomes, the atypical genomic composition detected by Alien_hunter, being flanked by a tRNA, as well as high SNP densities but low dN/dS ratios, and incongruent phylogenetic trees together provide evidence of HGT within the *ptx/ptl* loci (Figures 6 and 7). Expression of Ptx has not yet been observed in *B. bronchiseptica* or *B. parapertussis* strains; however, the low dN/dS ratio reflects purifying selection, indicating there are conditions under which these loci must be expressed. The facts that the entire operon is conserved in both *B. bronchiseptica* and *B. parapertussis*[48], and there is non-random distribution of SNP accumulations in different genes of this locus further support this view. Ptx is known for multiple biological activities, including both enzymatic activity on a range of different G proteins [49] and the ability to bind to various mammalian cells [50,51]. The ability to vary these activities to confront different hosts could be important to the broad host range of these organisms. In this light, the large number of amino acid substitutions in *ptxC* is intriguing. *ptxB* and *ptxC* are highly homologous and appear to have arisen via gene duplication. Since the proteins they encode are known to contain regions that determine binding specificity [50,51] and interact with extracellular receptors [52], the greater variation in these genes could contribute to adaptation to new hosts (see Additional file 8). Possible partial redundancy between the two gene products could allow diversifying without the cost of deleterious mutations necessarily resulting in complete loss of Ptx activity [48]. Despite their close relationship to *B. pertussis, B. bronchiseptica* complex IV strains have *ptx/ptl* loci that are either highly divergent (D445) or completely lost (MO149 and Bbr77), supporting the immune-mediated competition [53] or change of the requirement for the *ptx/ptl* loci in these strains. The extensive variation in SNP densities within the *ptx* locus, and the substantially different SNP densities of the *ptx* and *ptl* loci suggest that there are more complexities to the evolution of these lineages than can be explained by simple descent with variation and positive/negative selection.

## Conclusions

The current comparative genomic analysis of multiple classical *Bordetella* subspecies has revealed a complex and flexible pan-genome with limited introduction of new genetic material. Genome-wide SNP-based phylogenetic trees of the classical Bordetellae provide a robust model of their genetic relationship against which to measure the relative evolutionary pressures on the various factors that might affect their success differently in various environments. Evolution of virulence-associated genes appears to occur via mechanisms that include the random SNP accumulations as well as more directed mechanisms, such as HGTs, that can explain high SNP densities. Both of these or potentially more interesting novel mechanisms are likely to contribute to the substantial phenotypic diversity amongst the classical *Bordetella* subspecies. The importance of these organisms as pathogens of humans and other mammals as well as their recent evolutionary changes in critical pathogen characteristics raise the significance of understanding the genesis and effects of these diversity-generating mechanisms.

## Materials and methods

### Sequencing and assembly of genomes

The genomes of Bpp5 (accession number HE965803, plasmid accession number HE965804), 253 (accession number HE965806), MO149 (accession number HE965807), also known as D444, and 18323 (accession number HE965805) were sequenced to approximately 9-fold coverage (or 6-fold for 18323) by shotgun sequencing from two genomic shotgun libraries, pMAQ1Sac_BstXI (with insert sizes of 6-9 kb) and pOTWI2 (with insert sizes of 4-5 kb; 5-6 kb), that were sequenced using big-dye terminator chemistry on ABI3730 automated sequencers [20]. End sequences from large insert fosmid libraries in pCC1FOS with an insert size of 38-42 kb were used as a scaffold. This generated approximately 0.2- to 0.4-fold coverage. The assembly was generated using phrap2gap. All repeat regions and gaps were bridged by read-pairs or end-sequenced polymerase chain reaction (PCR) products that were again sequenced with big dye terminator chemistry on ABI3730 capillary sequencers. The sequences were manipulated to the ‘Finished’ standard for Bpp5, MO149, and 18323 or to the ‘Improved High Quality Draft’ standard for 253 [54]. The genomes of D445 (accession number HE983627) and Bbr77 (accession number HE983628) were sequenced on both the Illumina GAII platform (54 bp paired end library) and the Roche 454 machine using GSFLX Titanium chemistry (3 kb insert paired end library) [16]. The Illumina data were assembled using Velvet [55] at a k-mer value of 39 or 31, respectively. This assembly was then shredded and combined with 454 data using Newbler [17,18].

The genome of 1289 (accession number HE983626) was sequenced using the Roche 454 GSFLX sequencing platform [12,56] and assembled using Newbler [17,18], which resulted in 32-fold coverage and 133 contigs. The order of these contigs was determined by using RB50 as a reference genome. Gaps were closed by using sequences from PCR amplification of gap regions generated using big dye terminator chemistry on an ABI3730 capillary sequencer, and were assembled using PHRED, PHRAP, and CONSED [57-59], sequences generated from a 76 bp paired end library using a GAIIX [16] (resulting in 188-fold coverage), and sequences generated from a 3 kb insert paired end library using a GSFLX platform [17,18]. These long-tag paired end reads and optical maps [19] (OpGen, Gaithersburg, MD) were used as scaffolds to identify and correct misassemblies and verify the final assembly. SNP sequencing errors were corrected by mapping the sequences generated by the GAIIX to the final genome assembly using inGAP [60]. The final draft of the 1289 genome consists of seven ordered contigs, covering >99.99% of the genome.

### Annotation of genomes

Both RAST [61] and an automated annotation transfer tool at the Sanger Institute (*unpublished*) were used to annotate the finished or draft genomes of MO149, 1289, 253, D445, Bbr77, Bpp5, and 18323. The annotation tools were also used to annotate the *ptx/ptl* loci of six *B. pertussis* genomes (B0558, B1193, B1831, B1834, B1917, and B1920). We used RAST results for the novel regions that were not automatically transferred from the reference genomes, RB50, 12822, and Tohama I, based on their species classification. Each novel gene prediction was also curated with BLAST [62] and FASTA [63] results, and Pfam [64] and Prosite [65] were used to identify protein motifs. Transmembrane domains, signal sequences, and rRNA genes were identified with TMHMM [66], SignalP [67], and BLASTN [62], respectively. ISFinder was also used to detect Insertion Sequence (IS) elements in the genomes [68]. Manual curation was done with these novel regions using Artemis [69] and Artemis Comparison Tool (ACT) [70].

### Pan-genome analysis

Coding sequences were extracted from the eleven classical *Bordetella* genomes as well as *B. petrii* and *B. avium* genomes, and orthologous gene families were determined using OrthoMCL [22,23], which defines putative pairs of orthologs based on reciprocal all-against-all BLASTP [62] with a cutoff E-value of 10^-5^, over 70% length coverage, and at least 70% identity. The orthologs are then clustered using Markov cluster algorithm. When gene families were not clustered and had no BLAST hits, they were considered strain specific gene families. The results of OrthoMCL were converted to a pan-genome matrix profile for further analysis. A pan-genome matrix was constructed with each column as a genome and each row as a gene family. Cell *(i,j)* in the matrix is 1 when gene family “i” is present in genome “j”, or 0 when gene family is absent. A graphical representation of the classical Bordetellae pan-genome (Figure 1) was created using the Circos software [21].

The sequential inclusion of up to eleven strains (eleven columns in a pan-genome matrix) was simulated in all possible combinations (*N* = 11!/ [(n-1)!*(11-n)!]). The number of new gene families, the core genome, and pan-genome size were estimated using methods adapted from Tettelin *et al.*[26]. The R [71] function *nls* was used for non-linear least squares regression on the mean of the new gene families, core gene families, and pan-genome distributions. The number of expected new gene families and core gene families (*n*) determined by sequencing an *Nth* genome was modeled by the power law function *n* = *κN*^-*α*^, and the pan-genome size (*n*) by a power law *n* = *κN*^*γ*^. For estimation of novel gene families, the functions were fit to the mean values for all N ≥ 4, when there are more than 6 genomes to avoid the left side bias that could skew the results.

### Gene content tree

Hierarchical clustering was performed for the complete pan-genome matrix using pvclust in R [71], modified from Lukjancenko *et al.*[72]. The dendrogram was generated by hierarchical clustering with complete linkage method and Manhattan distances using R [71]. Bootstrap values were computed by resampling the rows of the matrix 1,000 times.

### Virulence factor comparison

Genes and the loci that encode the known virulence factors, filamentous haemagglutinin (FHA), fimbriae (Fims), pertactin (PRN), tracheal colonization factor (TcfA), invasive adenylate cyclase/haemolysin (ACT), dermonecrotic toxin (Dnt), pertussis toxin (Ptx), *Bordetella* resistance to killing (BrkA) [29], O-antigen [30], Type III secretion system (TTSS) [31], and Type VI secretion system (T6SS) [32], were compared among the eleven genomes via ACT [70]. Percent sequence similarity was calculated based on RB50 sequences with BLASTN [62], and genes that either contain a frame-shift mutation or an in-frame stop codon, or that are absent were highlighted with different colors in the heatmap that was generated by R [71]. The phylogenetic tree (Gene content tree) based on the presence and absence of each gene family in the pan-genome, excluding strain-specific genes, was superimposed on the heatmap.

### SNP analysis

All seventeen (*B. bronchiseptica* strains: 253, 1289, D445, Bbr77, and MO149, *B. parapertussis* strains: 12822 and Bpp5, *B. pertussis* strains: Tohama I, 18323, CS, B0558, B1193, B1831, B1834, B1917, and B1920, and *B. petrii* strain DSM 12804) genomic sequences were randomly shredded into 54 bp long reads and mapped onto the reference genome (RB50), using Ssaha v2.2.1 [73]. High quality candidate SNPs were identified using ssaha_pileup, and 128,752 SNP sites were identified in at least one strain based on RB50. Phylogenetic trees were constructed with RAxML v7.0.4 [74] for all SNP sites in the reference genome, using a General Time Reversible (GTR) model with a gamma correction for among site rate variation and ten random starting trees [75].

### dN/dS Analysis

The available *Bordetella* genomes were aligned onto the reference genome (RB50) using MAUVE aligner with default parameters [76]. Orthologous sequences were extracted from the alignments using gene coordinates defined in RB50 and processed to cover the entire coding regions of each individual genome, excluding pseudogenes. The nucleotide sequence alignments were later refined using their corresponding amino acid sequence alignments. dN and dS values were computed using PAML package [77] with the Nei-Gojobori method [78].

### HGT Detection

Putative horizontally acquired regions in the eleven classical *Bordetella* strains were predicted by Alien_hunter, which is able to detect genomic regions that may originate from foreign sources using Interpolated Variable Order Motifs (IVOMs) [41]. For the phylogenetic tree comparison, multiple alignments of individual genes and the entire *ptx/ptl* loci were generated by the MEGA5 software [79], and maximum likelihood trees were constructed with a Tamura-Nei model and 1,000 bootstrap replicates.

## Abbreviations

SNP: Single nucleotide polymorphism; HGT: Horizontal gene transfer; CGH: Comparative genome hybridization; MLST: Multi-locus sequence typing; PCR: Polymerase chain reaction; IS: Insertion sequence; GTR: General time reversible; IVOM: Interpolated variable order motifs; PAI: Pathogenicity island; Ptx: Pertussis toxin.

## Competing interests

The authors declare that they have no competing interests.

## Authors’ contributions

J. Park, YZ and AMB conceived and designed the research, performed the research, analyzed data, and wrote the manuscript; ETH conceived and designed the research, analyzed data, and wrote the manuscript; J. Parkhill and JFM conceived and designed the research; SCS conceived and designed the research as well as analyzed the data; UA and ML conceived and designed the research, analyzed the data, and contributed materials and reagents; MS analyzed the data and contributed analysis tools; XZ and SB performed the research. All authors read and approved the final manuscript.

## Supplementary Material

Additional file 1 **Comparative genome content of thirteen *Bordetella* strains.** Circles indicate the presence (solid color) or absence (unfilled) of each gene family in each strain examined. Circles from outer to inner are started with *B. petrii* strain followed by *B. avium* strain and the pan-genome of the classical *Bordetella* strains. Then, individual circle of *B. bronchiseptica*, *B. parapertussis* and *B. pertussis* strains were shown. This figure was created using the Circos software [21]. Click here for file

Additional file 2 **Lists of gene families that are present in the pan-genome of the classical *Bordetella* strains.** This table summarized the gene family numbers, representative gene name, predicted function of encoding proteins, presence (1) or absence (0) of each gene family in individual genome, and the sum of each row for a sorting purpose. Click here for file

Additional file 3 **The number of SNPs in the genes and dN/dS ratios of genes that have high number of SNPs.** This table summarized the start and end position of genes in RB50, gene name, strand, gene length, predicted function of encoding proteins, number of SNPs among the classical Bordetellae based on RB50, and number of SNPs per 100 base pair. dN, dS, and dN/dS ratio are included only for fifty genes that have high number of SNPs. The mean SNPs per 100 bp for the whole genome was 2.24. When the orthologs are pseudogenes, we did not calculate dN/dS ratios, indicating NA in the columns. Click here for file

Additional file 4 **Percent sequence similarity of *B. pertussis ptx/ptl* loci with flanking genes against Tohama I.** Percent sequence similarity of *B. pertussis ptx/ptl* loci and flanking genes compared to Tohama I was plotted between 95% and 100% using zPicture [36]. Intergenic regions, coding regions, and a tRNA were highlighted with red, blue, and green, respectively. Click here for file

Additional file 5 **Pair-wise dN/dS ratios for individual gene in the classical Bordetellae *ptx/ptl *locus.** dN/dS ratios of ten classical Bordetellae were plotted for individual gene in the *ptx/*ptl locus. *B. pertussis* strain Tohama I represents another *B. pertussis* strain CS as well because they share identical nucleotide sequences in the *ptx/ptl* loci. Each figure represents ptx*/ptl* genes (A), *ptx* genes (B), *ptxA* (C), *ptxB* (D), *ptxD* (E), *ptxE* (F), *ptxC* (G), *ptl* genes (H), *ptlA* (I), *ptlB* (J), *ptlC* (K), *ptlD* (L), *ptlI* (M), *ptlE* (N), *ptlF* (O), *ptlG* (P), and *ptlH* (Q). Click here for file

Additional file 6 **Genome-wide horizontal gene transfer candidates for the classical Bordetellae.** The position of the candidates is plotted in each genome (Bpp5 (A), 12822 (B), Tohama I (C), CS (D), 18323 (E), RB50 (F), 1289 (G), 253 (H), MO149 (I), Bbr77 (J), and D445 (K)) and the height represents the score of Alien_hunter. Red line represents the threshold for each genome. Click here for file

Additional file 7 **Phylogenetic trees for the classical Bordetellae *ptx/ptl* locus.** Phylogenetic trees were constructed by maximum likelihood method with 1,000 bootstrap replicates with individual gene sequences in the locus, or the entire *ptx* or *ptl* locus. Each figure represents the entire *ptx* locus (A), *ptxA* (B), *ptxB* (C), *ptxD* (D), *ptxE* (E), *ptxC* (F), *ptl* locus (G), *ptlA* (H), *ptlB* (I), *ptlC* (J), *ptlD* (K), *ptlI* (L), *ptlE* (M), *ptlF* (N), *ptlG* (O), and *ptlH* (P). Click here for file

Additional file 8 **Multiple sequence alignments of the classical Bordetellae *ptxB* and *ptxC*.** Multiple amino acid sequence alignments of the classical Bordetellae *ptxB* and *ptxC* were presented in this figure. Click here for file
