# Supplementary figures and images for: Comparative genomics of the classical Bordetella subspecies: the evolution and exchange of virulence-associated diversity amongst closely related pathogens
Source: BMC Genomics. 2012 Oct 10;13:545. doi: 10.1186/1471-2164-13-545 (PMC3533505; doi:10.1186/1471-2164-13-545)

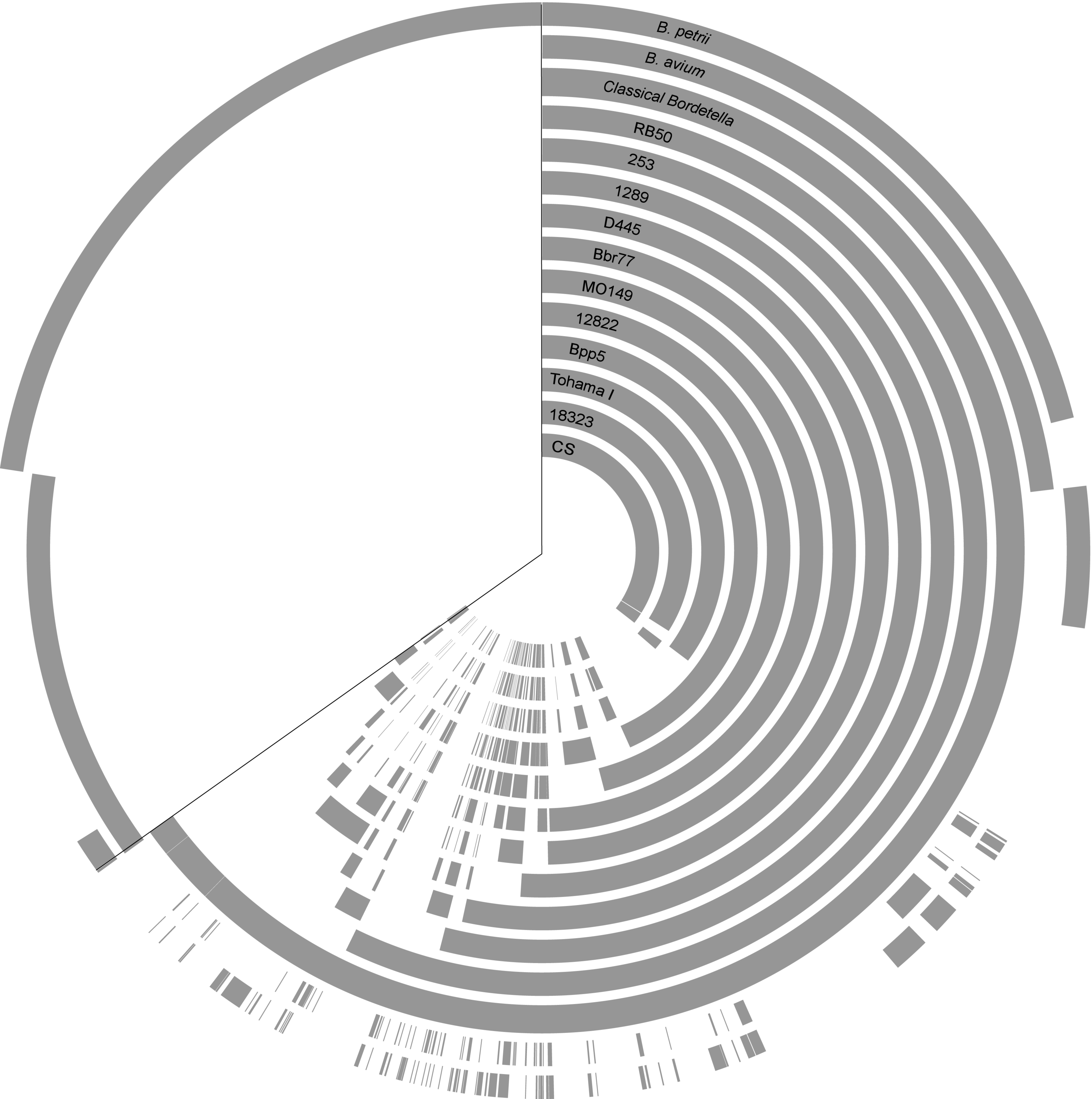

Supplement: Additional file 1 — Comparative genome content of thirteen Bordetella strains. Circles indicate the presence (solid color) or absence (unfilled) of each gene family in each strain examined. Circles from outer to inner are started with B. petrii strain followed by B. avium strain and the pan-genome of the classical Bordetella strains. Then, individual circle of B. bronchiseptica, B. parapertussis and B. pertussis strains were shown. This figure was created using the Circos software [21]. [file 1471-2164-13-545-S1.pdf]

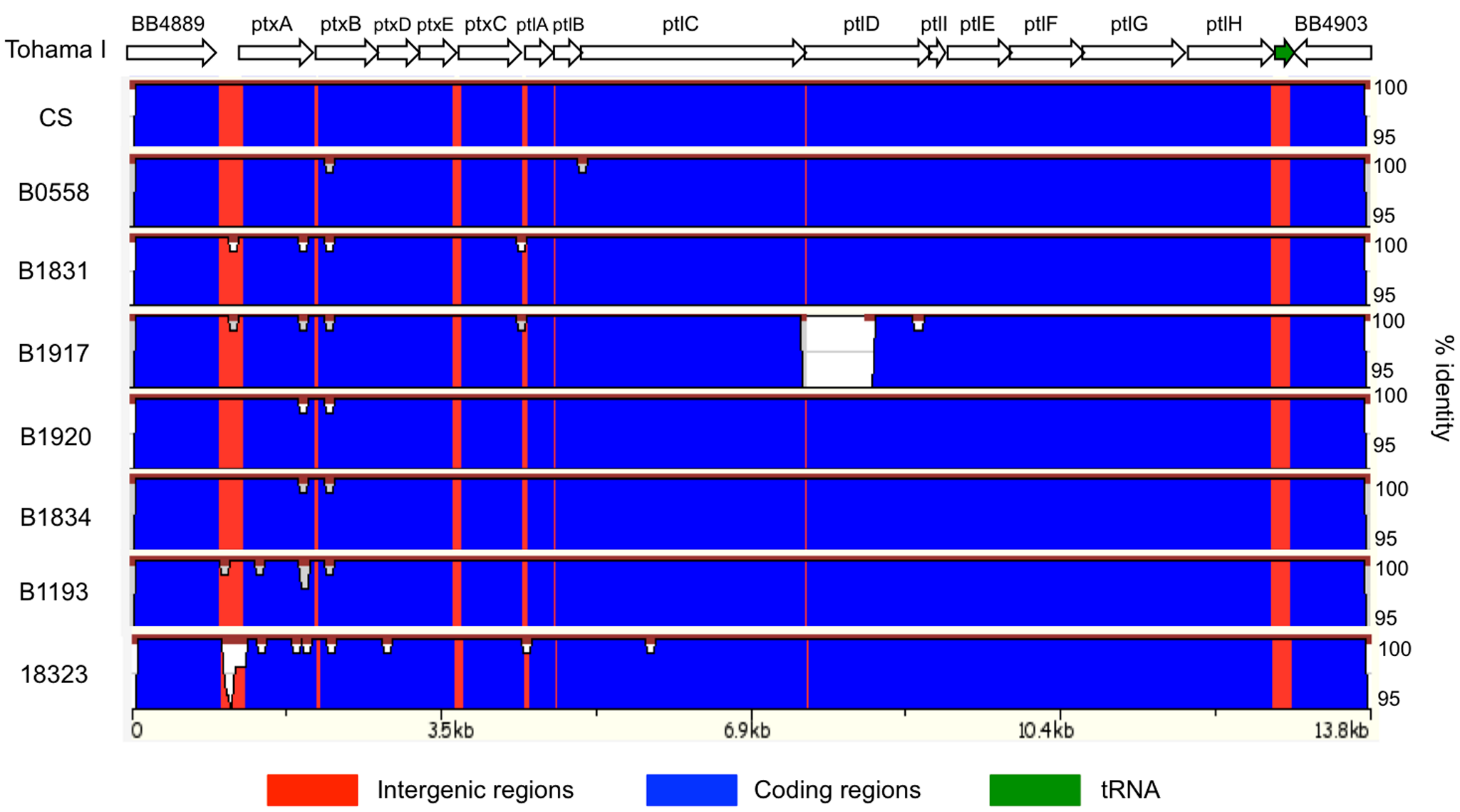

Supplement: Additional file 4 — Percent sequence similarity of B. pertussis ptx/ptl loci with flanking genes against Tohama I. Percent sequence similarity of B. pertussis ptx/ptl loci and flanking genes compared to Tohama I was plotted between 95% and 100% using zPicture [36]. Intergenic regions, coding regions, and a tRNA were highlighted with red, blue, and green, respectively. [file 1471-2164-13-545-S4.pdf]

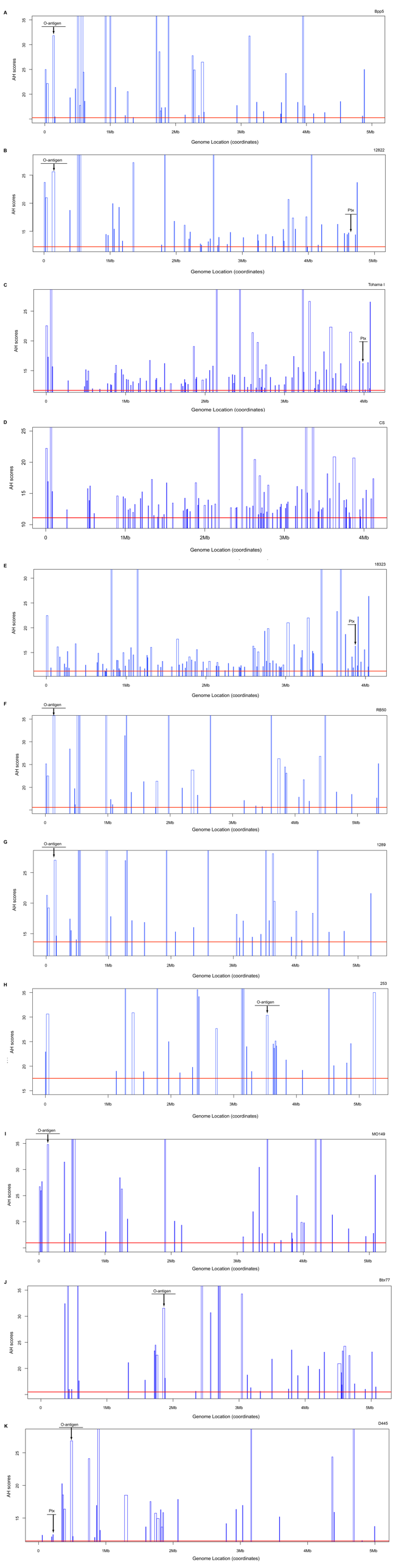

Supplement: Additional file 6 — Genome-wide horizontal gene transfer candidates for the classical Bordetellae. The position of the candidates is plotted in each genome (Bpp5 (A), 12822 (B), Tohama I (C), CS (D), 18323 (E), RB50 (F), 1289 (G), 253 (H), MO149 (I), Bbr77 (J), and D445 (K)) and the height represents the score of Alien_hunter. Red line represents the threshold for each genome. [file 1471-2164-13-545-S6.pdf]

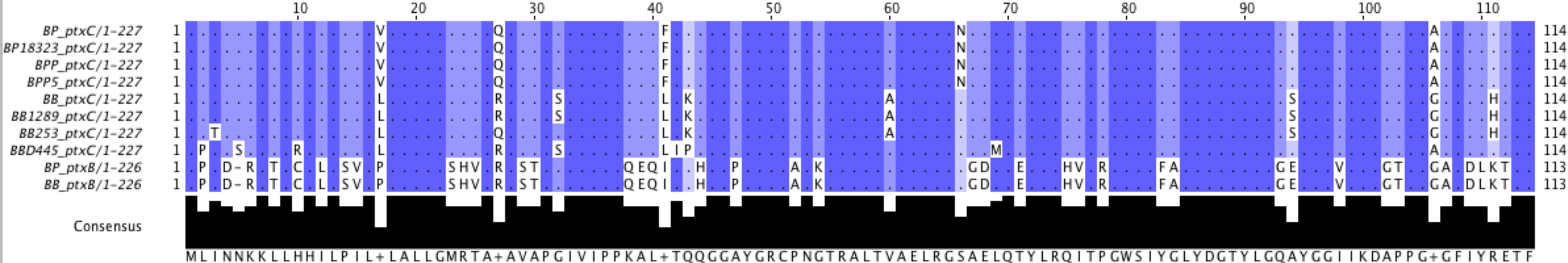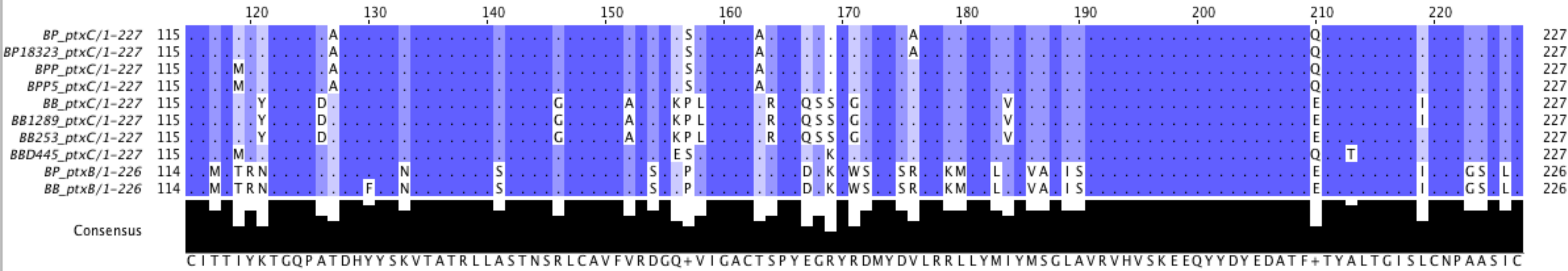

Supplement: Additional file 8 — Multiple sequence alignments of the classical Bordetellae ptxB and ptxC. Multiple amino acid sequence alignments of the classical Bordetellae ptxB and ptxC were presented in this figure. [file 1471-2164-13-545-S8.pdf]
