# Supplementary material for: Comparative genomics of the classical Bordetella subspecies: the evolution and exchange of virulence-associated diversity amongst closely related pathogens
Source: BMC Genomics. 2012 Oct 10;13:545. doi: 10.1186/1471-2164-13-545 (PMC3533505; doi:10.1186/1471-2164-13-545)
Supplement: Additional file 5 — Pair-wise dN/dS ratios for individual gene in the classical Bordetellae ptx/ptl locus. dN/dS ratios of ten classical Bordetellae were plotted for individual gene in the ptx/ptl locus. B. pertussis strain Tohama I represents another B. pertussis strain CS as well because they share identical nucleotide sequences in the ptx/ptl loci. Each figure represents ptx/ptl genes (A), ptx genes (B), ptxA (C), ptxB (D), ptxD (E), ptxE (F), ptxC (G), ptl genes (H), ptlA (I), ptlB (J), ptlC (K), ptlD (L), ptlI (M), ptlE (N), ptlF (O), ptlG (P), and ptlH (Q). [file 1471-2164-13-545-S5.pdf]

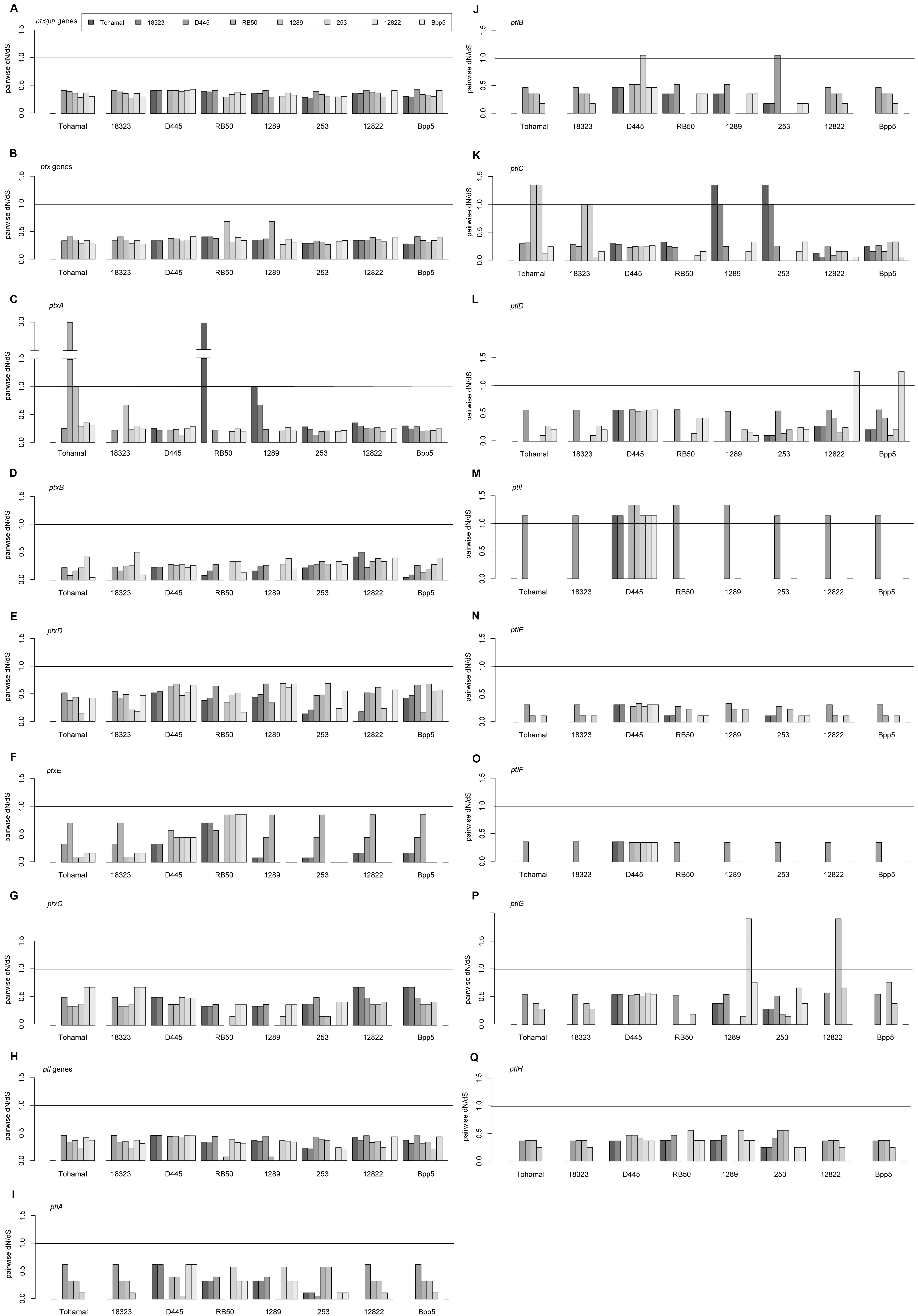
A
ptx/ptl genes
Tohamal
18323
D445
RB50
1289
253
12822
Bpp5
pairwise dN/dS
B
ptx genes
C
ptxA
D
ptxB
E
ptxD
F
ptxE
G
ptxC
H
ptl genes
I
ptlA
J
ptlB
K
ptlC
L
ptlD
M
ptlI
N
ptlE
O
ptlF
P
ptlG
Q
ptlH
