# Supplementary material for: Comparative genomics of the classical Bordetella subspecies: the evolution and exchange of virulence-associated diversity amongst closely related pathogens
Source: BMC Genomics. 2012 Oct 10;13:545. doi: 10.1186/1471-2164-13-545 (PMC3533505; doi:10.1186/1471-2164-13-545)
Supplement: Additional file 7 — Phylogenetic trees for the classical Bordetellae ptx/ptl locus. Phylogenetic trees were constructed by maximum likelihood method with 1,000 bootstrap replicates with individual gene sequences in the locus, or the entire ptx or ptl locus. Each figure represents the entire ptx locus (A), ptxA (B), ptxB (C), ptxD (D), ptxE (E), ptxC (F), ptl locus (G), ptlA (H), ptlB (I), ptlC (J), ptlD (K), ptlI (L), ptlE (M), ptlF (N), ptlG (O), and ptlH (P). [file 1471-2164-13-545-S7.pdf]

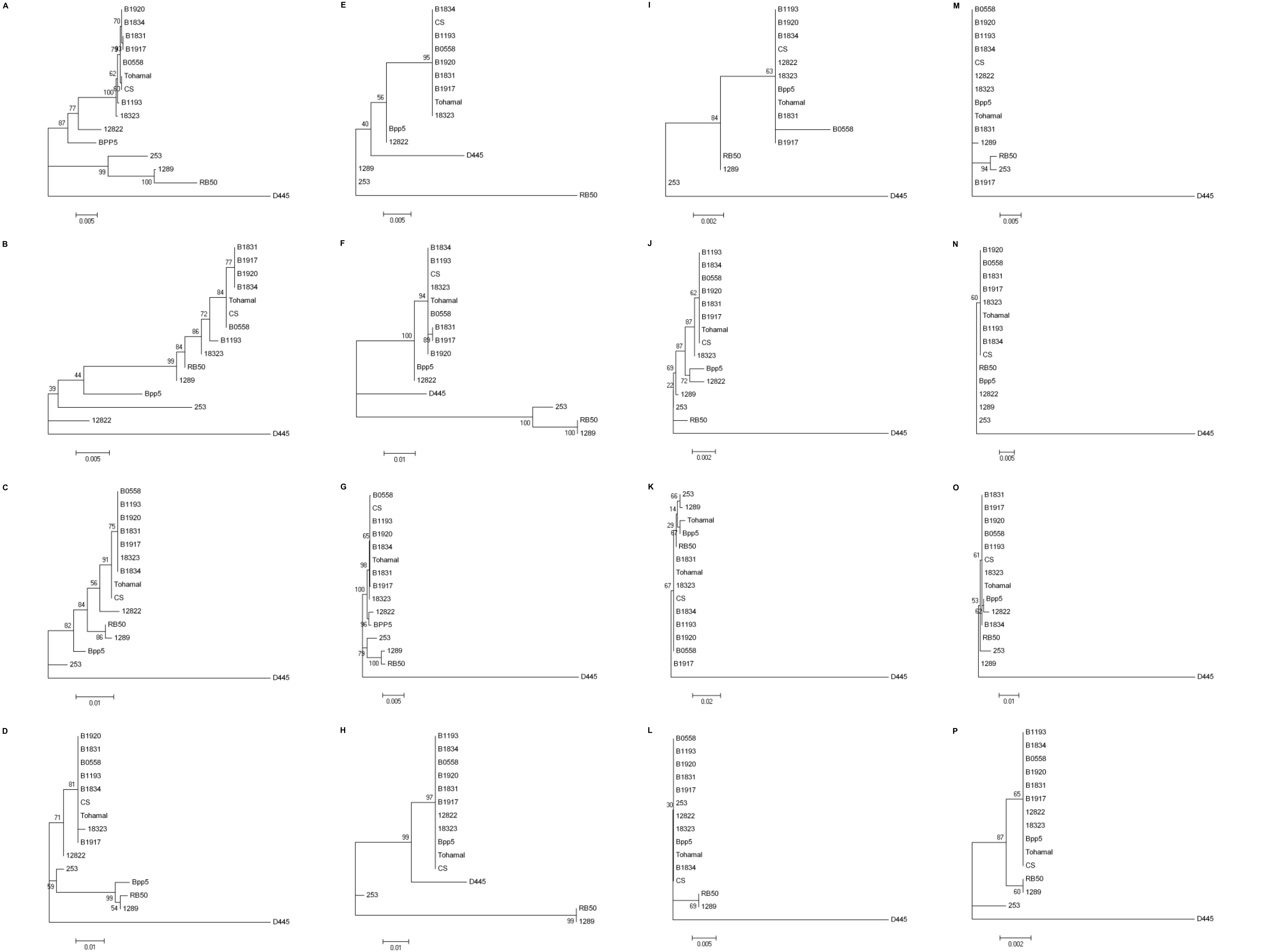
A
B1920
70
B1834
B1831
79
93
B1917
B0558
62
Tohamal
100
60
CS
B1193
18323
77
87
12822
BPP5
253
99
1289
100
RB50
D445
0.005
B
B1831
77
B1917
B1920
B1834
84
Tohamal
72
CS
B0558
86
B1193
84
18323
99
RB50
1289
44
Bpp5
39
253
12822
D445
0.005
C
B0558
B1193
B1920
75
B1831
B1917
91
18323
B1834
56
Tohamal
CS
84
12822
82
RB50
86
1289
Bpp5
253
D445
0.01
D
B1920
B1831
B0558
B1193
81
B1834
CS
Tohamal
71
18323
B1917
12822
253
59
Bpp5
99
RB50
54
1289
D445
0.01
E
B1834
CS
B1193
B0558
95
B1920
B1831
B1917
Tohamal
18323
56
40
Bpp5
12822
D445
1289
253
RB50
0.005
F
B1834
B1193
CS
18323
94
Tohamal
B0558
100
B1831
89
B1917
B1920
Bpp5
12822
D445
253
100
RB50
100
1289
0.01
G
B0558
CS
B1193
65
B1920
B1834
Tohamal
98
B1831
100
B1917
18323
12822
96
BPP5
253
79
1289
100
RB50
D445
0.005
H
B1193
B1834
B0558
B1920
B1831
97
B1917
12822
18323
99
Bpp5
Tohamal
CS
D445
253
RB50
99
1289
0.01
I
B1193
B1920
B1834
CS
12822
63
18323
Bpp5
Tohamal
84
B1831
B0558
B1917
RB50
1289
253
D445
0.002
J
B1193
B1834
B0558
62
B1920
B1831
B1917
87
Tohamal
87
CS
18323
69
Bpp5
72
12822
22
1289
253
RB50
D445
0.002
K
66
253
14
1289
29
Tohamal
87
Bpp5
RB50
B1831
Tohamal
67
18323
CS
B1834
B1193
B1920
B0558
B1917
D445
0.02
L
B0558
B1193
B1920
B1831
B1917
30
253
12822
18323
Bpp5
Tohamal
B1834
CS
RB50
69
1289
D445
0.005
M
B0558
B1920
B1193
B1834
CS
12822
18323
Bpp5
Tohamal
B1831
1289
RB50
94
253
B1917
D445
0.005
N
B1920
B0558
B1831
B1917
60
18323
Tohamal
B1193
B1834
CS
RB50
Bpp5
12822
1289
253
D445
0.005
O
B1831
B1917
B1920
B0558
B1193
61
CS
18323
Tohamal
53
Bpp5
62
12822
B1834
RB50
253
1289
D445
0.01
P
B1193
B1834
B0558
B1920
B1831
65
B1917
12822
18323
87
Bpp5
Tohamal
CS
RB50
60
1289
253
D445
0.002
